# An Essay on the Structure and Functions of the Skin

**Published:** 1833-04-01

**Authors:** 


### AN ESSAY ON THE STRUCTURE AND FUNCTIONS OF THE SKIN. By *W. Wood*, M.D. Octavo, pp. 172.

THIS is a very intelligent and useful summary of what is known at present on cutaneous physiology ; it contains, indeed, little or no new matter, excepting a few experiments on the function of perspiration from the skin and lungs ; but as it is of use to have known and established facts, or even what are considered as such, frequently presented to the memory, we shall spare a few lines to mark down some of the leading phenomena which have been observed by the best physiologists. The experiments of Lavoisier and Seguin are very generally considered to be worthy of much credit, on the subject of the cutaneous and pulmonary perspirations; they state that the average quantity lost in 24 hours, is 18 ozs. from the lungs, and 36 ozs. from the skin.

Cruickshank and Abernethy inferred, from some trials in which they kept their hands or their arms for an hour in air-tight vessels, and there ascertained the increase of the weight which the vessels acquired, that the quantity of the skin perspiration was about 2½ pounds in 24 hours. Dr. Wood has repeated many of the experiments, and gives a higher estimate; he supposes the quantity to amount to 45¾ ozs. in 24 hours. The estimate given by different authors of the quantity of the pulmonary exhalation have been equally discordant. Hales rates it at 20 ozs.—Abernethy at 9 ozs.—Dr. Thomson, of Glasgow, at 19 ozs.—Menzies at 6 ozs.—our author at 6 ozs. On the much-disputed question, whether the skin absorbs or not? Dr. Wood very justly employs the analogical affirmative argument, to be drawn from the skin of lizards and of the other batrachia possessing indisputably the power of absorption, and that, too, in a high degree (see our review of Dr. Edwards on Life, in our last No.) Nor are we to reject the evidence of Capt. Bligh and others, on the astonishing and most pleasing effects of wetting the surface to allay the thirst and burning, when their fresh water had failed them. Dr. Wood remarks that—

"It is very much to be desired that some experiments were made, in order to determine how far cutaneous absorption, of the kind under consideration, is connected with the properties termed *endosmose* and *exosmose*, discovered by Dutrochet to belong to organized membranes. Since, according to this discovery, when an organized membrane is interposed between two fluids of different densities, there arises a much more copious determination of the thinner fluid through the membrane towards the thicker, than of the thicker towards the thinner, is it not possible that the nature of the fluid placed in contact with the skin, as denser or rarer than the blood, or than the serum, may be a fertile cause of variation in the result of experiments, such as are mentioned above?" 53.

That a very rapid absorption of moisture from the atmosphere, either by the lungs or by the skin, takes place in many cases of diabetes, is abundantly proved by the body not losing weight in proportion to the great excess of the egesta over the ingesta;—thus Dr. Dill reports a case, in which the patient, at the commencement of the disease, weighed 145lbs; at the end of five weeks he weighed only 27lbs. less, although the daily excess of urine over the ingesta averaged five pounds; so that 113lbs. of urine must be accounted for by absorption from the atmosphere. The question then arises, does this great absorption chiefly take place at the skin or in the lungs? The much greater extent of the mucous surface of the latter over the former, (calculated by Dr. Monro, 2ndus, to be at least 30 or 40 times as great,) might lead us to suppose that the lungs are the principal organs of absorption; but several potent objections may be stated to this view; thus we know that expired air is in general saturated with moisture, as may be ascertained by merely breathing on a glass, especially in cold weather, which condenses the humidity more quickly; and as this is the case, how can we believe that rapid absorption is going on at the same time from the same surface? Again, the quantity of air exposed to the action of the pulmonary surface in the course of the day must be much smaller than of that which passes in contact with the skin; for not only is the number of inspirations limited, but also the air, when once admitted into the lungs, is confined there, till expelled by expiration; whereas at the skin, there is a constant and unceasing removal of the air, which thus becomes the vehicle for more copious absorption, as well as of more copious exhalation. The remarks of Dr. Wood are so pertinent, that they deserve to be transcribed.

"The very small extent of the skin, as compared with the extent of the bronchial membrane, raises, at first sight, a very strong objection to this assumption. But the quantity of air, and consequently the quantity of moisture, which comes in contact with the skin, in the course of a day, exceeds that which is received into the lungs in a much higher ratio, than the dimensions of the bronchial membrane exceed those of the skin. The great difference between the quantities of air brought to act on these two membranes, in a given time, will appear clearly, by a reference to the difference in the daily watery exhalation of each. The bronchial membrane, so many times more extensive, so plainly adapted to copious secretion, does not yield more than six ounces of watery exhalation to the air in twenty-four hours, while the skin, so moderate in size, and to appearance so little calculated for copious secretion, yields six times as much, or thirty-six ounces, in the same period. No other cause can be assigned for this difference, but that the air received into the lung, is so limited in quantity, that, notwithstanding its great rise of capacity for moisture, it can take up only this small proportion of the amount, which the ever-changing air, at the surface, carries away with very little addition to its capacity." 61.

Even when we admit that the skin may be better fitted than the surface of the air-cells for the absorption of humidity, the objection may readily suggest itself, is it possible that the air, which is at every moment abstracting moisture from a surface, can, at the same time, yield moisture to that surface, in very considerable quantity? We answer, yes; and we assert, that evaporation is constantly going on from wet inanimate bodies, even into an atmosphere loaded with moisture. If we take a deliquescent salt, and expose it for some time, it will become quite liquid even in a dry atmosphere; yet it cannot be fairly doubted, that an evaporation goes on from the very first deposition of moisture, just as freely as from any of the surrounding moist or wet bodies, and that the final accumulation of water is not the real measure of absorption, but merely the difference between the entire quantity absorbed, and what had been exhaled in the mean time. If this be granted, it is easily understood how the two processes may be going on at the surface of the skin, and we know that the cuticle is well adapted for the imbibition of moisture, its structure and composition being almost identical with those of the human hair, which has been proposed by Saussure as a delicate hygrometer.

On the subject of the rete mucosum, the various opinions of different authors are shortly and explicitly stated. It is worth while to record what the distinguished comparative anatomist has written regarding it.

"The vascular net-work which is situated immediately over the cutis, occupying its whole surface, is in general of an exceedingly thin texture; it is formed entirely of arterial, venous, and lymphatic vessels, which undergo many complex ramifications and anastomoses. This net-work is spread over the projections situated on the surface of the cutis. The pigment does not perhaps exist in all animals: it forms, at the surface of the vascular net-work, a layer, more or less defined, of slight consistence, semi-fluid, and in effect composed entirely of very minute grains, agglutinated to each other, without any organic continuity between their own particles, or with the other portions of the skin. It is a sort of artificial membrane or depository, which is variously coloured, and which seems to be exhaled by the parietes of the veins. This pigment, and the vascular net-work, are both crossed by the nervous extremities, which meet at the surface of the skin, sometimes under the form of papillæ. These two parts of the skin are those which, since the time of Malpighi, have been known by the name of Malpighi's net-work, *corpus reticulare reticulum mucosum*, on account of the sort of net-work they form, for the passage, not only of the mucous papillæ, but also of the accessory parts. They are both, in my opinion," he continues, "the source of the colouring matter, and the pigmentum is the depository of that matter."* 71.

Lieutaud and Cruickshank were the first who maintained that the rete mucosum is composed of two separate layers.

"When a blister," says Cruickshank, "has been applied to the skin of a ne-

---

* See De Blainville De l'Organization des Animaux.

gro, if it has not been very stimulating, in twelve hours after, a thin, transparent greyish membrane is raised, under which we find a fluid. This membrane is the cuticle, or scarf-skin. When this, with the fluid, is removed, the surface under these appears black ; but if the blister had been very stimulating, another membrane, in which this black colour resides, would also have been raised with the cuticle. This is the *rete mucosum*, which is itself double, consisting of another greyish transparent membrane, and of a black web, very much resembling the *pigmentum nigrum* of the eye."* 73.

We cannot afford space to follow our author into the digression on the varieties of the human race; although interesting, it is certainly quite misplaced in an essay which professes to treat only of the structure and functions of the skin, in the states of health and of disease. The work, however, is intended, we are told, for the general, as well as for the professional reader.

The second section is devoted to the consideration of the agency of the skin in the production of diseases. It is very generally known, that there is a mutual balance, and equilibrium of compensation between many of the functions of the body, especially between those of the skin, and of the kidneys, lungs and liver. The following remarks are good.

"The degree to which a variation in the supply of blood to one organ can take place without detriment to health, is much extended by that species of sympathy or conservative balance before remarked as existing among the several secretions. Thus, when the determination of blood to the skin is diminished by a cold atmosphere, and the secretion in consequence decreases, an augmented secretion by the kidneys generally arises, so as to prevent the mischiefs which might otherwise result from the accumulation, on the internal organs, of the fluids repelled from the surface. And in some cases of this kind, as was shewn in the foregoing experiments, the watery exhalation by the lungs becomes more abundant when the cutaneous secretion is diminished. Nay, farther, there is great reason to suppose that it is by this same power of accommodation among the secretions, carried to a much greater extent, that the human body is fitted to exist in every variety of climate. Thus, in tropical countries, where the activity of the skin becomes permanently augmented, the kidney becomes a less efficient organ—secreting not merely less watery fluid, but even, if we may judge from the almost complete exemption which the inhabitants of tropical regions enjoy from calculous disorders, ceasing to secrete the sparingly soluble saline matters, by the concretion of which calculi are generated." 115.

And again :—

"It has been ascertained, that the deposition of lithic acid from the urine is greater when the cutaneous perspiration is defective, and that, after copious perspiration, it becomes much less than usual. The perspiration, as was shewn above, is at all times acid ; and, though it has not been proved ever to contain lithic acid, it is reasonable to suppose with Dr. Philip, that, in copious perspiration, that acid is thrown off by the skin, or at least, that the necessity for so abundant a formation of it is superseded, by the larger separation of the proper acid of the cutaneous secretion. Yet, suppose this explanation little satisfactory, the above facts themselves agree well, both with the exemption of residents between the tropics, and with the known liability to stone and gravel of such individuals, in temperate countries, as have a scanty secretion by the skin. A practical inference of much importance is obtained from these observations,—the

* Experiments on the Insensible Perspiration.

utility of a strict attention to keeping up a free secretion from the surface, in persons threatened with calculous complaints." 116.

When cold and moisture are united in the atmosphere, the cutaneous perspiration is diminished in a twofold manner; namely, by the constrictive effect of the cold on the skin, and by the smaller aptitude of the air to take up fluid. And, in this case, there is not the same disposition to re-action as when the cold is dry; principally, as it appears, because the energy of the respiration is impaired by the dampness of the air, which prevents the lungs from throwing off the ordinary amount of watery exhalation. Hence results the well-known prejudicial effects of a cold damp atmosphere on the health. We have long been satisfied that the agency and influence of the functions of the skin, over those of many internal organs, have been neglected and overlooked too frequently in medical practice; and, therefore, forcibly recommend the propriety of enquiry into the state of the cutaneous secretion, as well as of the pulmonary, and of the gastric, biliary, intestinal or urinary discharges, in all cases, especially of chronic disease. In obstinate dyspepsia, in diarrhœa, in atrophy and mesenteric obstructions, in calculous complaints, in phthisical and hæmoptoic tendencies, and in a host of others, not to speak of those which are strictly cutaneous, we have derived the very happiest effects from the continued use of baths, affusion, or sponging, and have been thus enabled, by attention to diet at the same time, to supersede the necessity of much internal medicine. We cannot dilate upon this important topic at present; but shall content ourselves by directing the thoughts of our readers to the very intimate sympathy and connexion between the states of the skin and of the gastro-intestinal surface, as strikingly displayed by the phenonena of that singular disease "pellagra." They will find some good illustrative cases, and some useful remarks, in the Periscope of our last Number.

